# Standardizing NIR spectroscopy for PAT in phytopharmaceutical applications: multivariate detection and quantification limits of vitexin and isovitexin

**DOI:** 10.1007/s00216-025-06209-z

**Published:** 2025-11-21

**Authors:** Krzysztof B. Beć, Justyna Grabska, Jan-Clemens Cremer, Christian W. Huck

**Affiliations:** Institute of Analytical Chemistry and Radiochemistry, University of Innsbruck, Innsbruck, Austria

**Keywords:** Process analytical technology (PAT), Process understanding, Multivariate LOD, NIR spectroscopy, Phytopharmaceuticals, Net analyte signal (NAS)

## Abstract

Near-infrared (NIR) spectroscopy, in combination with multivariate calibration, plays a central role in process analytical technology (PAT) for quantitative analysis. While detection and quantification limits (LOD/LOQ) are critical performance indicators, their estimation in multivariate models remains non-trivial—especially in complex systems such as phytopharmaceuticals. This study compares two multivariate LOD/LOQ estimation frameworks: the variance-leverage based mLOD/mLOQ (IUPAC-compliant, geometry-driven) and the net analyte signal (NAS)–based approach, which quantifies analyte-specific signals orthogonalized against the model background. Partial least squares regression (PLS-R) models were developed for vitexin and isovitexin in phytopharmaceutical samples of *Passiflora incarnata*. The study included four NIR spectrometers (benchtop and handheld) implementing various optical principles, as well as two sample conditions (milled, intact). LOD/LOQ estimates were systematically evaluated against the influence of latent variable structure, model complexity, and orthogonal signal correction (OSC), a spectral preprocessing technique that explicitly enhances the isolation of analyte-specific variance. The results demonstrate how mLOD and NAS-LOD respond to model geometry when applied to phytopharmaceutical matrices. Both frameworks explicitly depend on the latent structure constructed in the PLS-R model; however, NAS-LOD is highly sensitive to analyte signal alignment and dispersion across latent variables, while mLOD reflects variance-weighted geometric detectability. OSC pretreatment improved the analyte signal concentration in early latent variables and led to markedly reduced NAS-LOD values, while mLOD values showed substantial responsiveness to latent space compression in the models. The combined use of both frameworks, supported by latent space diagnostics (analyte variance per LV, projection of pure standards), provides a multi-layered evaluation of model transparency and internal structure. These findings demonstrate that multivariate LOD/LOQ estimation can reinforce PAT-oriented method development not only as a validation metric, but as a diagnostic tool that provides standardized measures for controlling the NIR analytical method.

**Graphical abstract:**

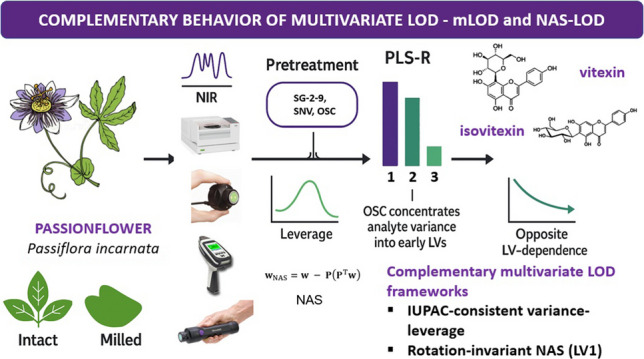

**Supplementary Information:**

The online version contains supplementary material available at 10.1007/s00216-025-06209-z.

## Introduction

The application of process analytical technology (PAT) concepts in pharmaceutical analysis has led to a paradigm shift in quality control, moving from end-product testing towards continuous monitoring of critical quality attributes (CQAs) during production [1, 2]. Among the available spectroscopic techniques, near-infrared (NIR) spectroscopy has gained particular importance due to its non-destructive nature, rapid data acquisition, and minimal sample preparation requirements [3, 4]. This is especially relevant for pharmaceutical applications, where real-time or inline analysis is essential for ensuring product consistency and regulatory compliance [5]. While its implementation in synthetic pharmaceutical production is well established [6, 7], the transfer of NIR spectroscopy to phytopharmaceutical applications introduces several additional analytical challenges [8–11]. These challenges arise from the complex and variable composition of plant-derived materials and frequent chemical interactions between analyte and matrix components—all factors that complicate model calibration, validation, and the reliable estimation of detection capability. International regulatory authorities, including the US Food and Drug Administration (FDA) and the European Medicines Agency (EMA), emphasize not only process control but also the transparency and interpretability of pharmaceutical analytical methods [12, 13]. The FDA guidance document explicitly encourages the use of real-time process monitoring and multivariate analytical methods to maintain product quality in pharmaceutical manufacturing processes [14]. Similarly, the EMA promotes PAT within its quality by design (QbD) framework, explicitly highlighting the importance of process understanding and control through multivariate data analysis and in-process measurements [15]. In this regulatory context, the interpretability of models—i.e., understanding how they capture, represent, and respond to process-relevant variance—is increasingly viewed as a critical complement to predictive accuracy.

Compared to synthetic pharmaceutical products, phytopharmaceutical materials are characterized by a markedly higher degree of variability at multiple levels of composition (e.g., intrinsic plant matrix, moisture) and physical properties (e.g., particle size distribution). The sample complexity has a clear consequence, making the latent structure in the models more convoluted and mixing the analyte variance with that originating from the matrix. These conditions impose significant challenges for multivariate calibration, increasing the model complexity and reducing its interpretability [16, 17]. In this context, strategies that not only mitigate overfitting but actively isolate and expose analyte-specific variance become particularly important for PAT-oriented model development in phytopharmaceutical analysis [18].

In rigorous analytical applications of spectroscopy, the ability to estimate detection and quantification limits (LOD/LOQ) in multivariate calibration models gains particular relevance. Classical univariate definitions of LOD and LOQ, typically based on signal-to-noise ratios or the residual standard deviation of blank measurements, are not directly transferable to multivariate models such as partial least squares regression (PLS-R). This requires adapted approaches that consider the internal arrangement of the calibration model, the distribution of analyte-relevant variance within the latent variable (LV) space, and the interaction between analyte signal and matrix background. Several methodological frameworks have been proposed to address this need. Ostra et al. proposed an initial extension of IUPAC logic into multivariate calibration, defining a variance-geometry-based multivariate LOD estimation [19]. A similar concept was subsequently formalized into a widely adopted strategy as a rigorous mLOD framework designed to be consistent with IUPAC principles. Introduced by Olivieri and co-workers [20–23], it defines multivariate LOD and LOQ (mLOD/mLOQ) based on instrumental variance, calibration variance, and the statistical leverage of the blank sample within the latent variable space, capturing the geometric and variance-based detectability of the analyte. An alternative perspective is offered by the net analyte signal (NAS)–based framework [24, 25], in which LOD and LOQ are linked to the magnitude of the analyte-specific component in the model latent structure, explicitly orthogonalized against background variance. These frameworks follow fundamentally different conceptual philosophies and, as a result, behave distinctly and capture non-equivalent aspects of model response. While mLOD reflects detectability in terms of model geometry and variance captured in the calibration, NAS-LOD emphasizes analyte delineation and latent structure interpretability, all of which are of critical importance in PAT-oriented modelling.

While both variance-leverage mLOD and NAS-LOD have been applied in general multivariate calibration contexts, their use in PAT settings—particularly for phytopharmaceutical products—remains rare. Prior studies have largely focused on methodological development or proof-of-concept evaluations in simplified systems [26]. Inagaki et al. investigated physical and instrumental factors influencing LOD in NIR spectroscopy (e.g., path length, light intensity, co-addition time) using potassium hydrogen phthalate solutions [27]. However, direct applications of either mLOD or NAS-LOD to highly variable, chemically complex phytopharmaceutical matrices within PAT-oriented workflows have so far been scarcely documented.

In the context of PAT, detection limits provide essential methodological information beyond predictive performance. They also indicate how well the analyte signal is represented within the model, contributing to method control and interpretability. Predictive error metrics (e.g., root mean square errors of cross-validation/prediction RMSECV/RMSEP) quantify overall prediction accuracy, delivering a direct operational performance—an important but single-dimensional information blind to where analyte variance resides in the model. Multivariate LOD/LOQ frameworks add a deeper layer of method evaluation by estimating the intrinsic detectability of the analyte as shaped by the internal model structure; however, they operate in a distinct and complementary manner. The variance-leverage mLOD reflects detectability as a structurally grounded estimate, less directly connected to analyte delineation per se. In contrast, NAS-LOD operates explicitly at the interpretability level, quantifying how well analyte variance is isolated from matrix and background contributions. By measuring the geometric orthogonalization of the analyte signal against latent components unrelated to **y**, NAS-LOD delivers direct structural insight into signal separation. This is particularly valuable in systems with strong matrix interference, such as phytopharmaceuticals, where analyte delineation and transparency are essential for regulatory robustness [28].

Consequently, the availability of distinct LOD and LOQ frameworks in multivariate PAT calibration provides additional layers of process understanding and method control. However, these frameworks are not yet standardized in practical analytical workflows. They remain conceptually distinct, behave differently, and respond in specific ways to both the original data structure and the applied pretreatment strategies. This is particularly evident for preprocessing methods that enforce orthogonalization of background variance, such as orthogonal signal correction (OSC) [29–31] that should, by definition, have a pronounced impact on NAS-LOD estimates, while influencing variance-leverage mLOD to a lesser extent.

Establishing a deeper understanding of these mechanisms, and systematically exploring their applicability and limitations in real phytopharmaceutical PAT scenarios, is essential for the standardization and methodological advancement of LOD/LOQ estimation in multivariate analysis. The present work addresses these aspects by comparing two fundamentally different LOD/LOQ estimation strategies—variance/geometry-driven mLOD and NAS-LOD—in PLS-R-based quantification of natural active pharmaceutical ingredients (APIs) in phytopharmaceutical products. Particular attention is given to their dependence on model complexity, latent structure, and preprocessing strategies, especially the role of OSC in shaping analyte delineation within the model. Vitexin and isovitexin were selected as analytical targets due to their pharmacological relevance and challenging analytical characteristics [32]. Both compounds are C-glycosylated flavones commonly occurring together in phytopharmaceutical products, especially in preparations based on *Crataegus* spp. (hawthorn) and *Passiflora* spp. (passionflower) [33, 34]. Their chemical similarity, spectral overlap, low concentration levels (typically low mg/g range), and occurrence within chemically and physically variable plant matrices create an analytically demanding scenario highly representative for PAT-oriented modelling in phytopharmaceutical settings. This study specifically focuses on *Passiflora incarnata* (passionflower), a medicinal plant of recognized phytopharmaceutical relevance [35, 36]. *Passiflora* spp. bear practical significance in contemporary phytotherapy, with standardized extracts widely used for their anxiolytic and sedative properties. Given the typically low concentrations of vitexin and isovitexin in such preparations, robust quantification demands particular attention to detection capability and model structure. This dual challenge defines the focus of the present study from both a methodological and a phytopharmaceutical quality control perspective.

The study systematically investigates how the choice of LOD/LOQ framework interacts with model behaviour, latent structure organization, and data pretreatment—with particular emphasis on method interpretability and analyte signal exposure. Special attention is given to the methodological implications of these findings for PAT applications, both in phytopharmaceutical and broader pharmaceutical contexts, highlighting critical aspects to be considered in future efforts towards standardization within the industry.

## Materials and methods

### Samples

The study was conducted on a total of 50 samples of *Passiflora incarnata* L. (passionflower) material, representative of typical phytopharmaceutical raw materials. The samples were collected from various commercial sources, including pharmacies, online suppliers, and herbal product manufacturers, with the intention to cover a wide range of batch-to-batch and supplier-dependent variability.

The plant material was analyzed both in milled and whole (intact) forms to reflect possible differences in process scenarios. Milled samples were prepared using a ZM 200 laboratory mill (Retsch GmbH, Haan, Germany) equipped with a 0.5-mm sieve; thorough milling was performed to obtain a well-homogenized sample. The HPLC dataset was used as reference (section) for both milled (ground) and intact samples in the NIR analysis. Each sample was subsequently measured by NIR spectroscopy in both physical states (intact and milled) using multiple instruments differing in technological class and miniaturization level, as detailed in the section.

### High-performance liquid chromatography

Reference analysis of vitexin and isovitexin content in the investigated samples was performed using high-performance liquid chromatography (HPLC) with UV detection (Shimadzu LC-2040C, Kyoto, Japan). Chromatographic separation was achieved on a Waters XSelect C-18 column (3.5 µm, 4.6 × 100 mm) operated at 30 °C, using reversed-phase gradient elution with 0.1% formic acid in water (eluent A) and acetonitrile (eluent B) at a flow rate of 0.8 mL/min. The injection volume was 10 µL. Detection was carried out at 337 nm using a Shimadzu LC-2040C system (Kyoto, Japan). Retention times for vitexin and isovitexin were approximately 6.0 and 6.8 min, respectively, with a resolution of Rs = 3.04 between both analytes, demonstrating reliable quantification. Sample preparation involved ultrasound-assisted extraction with 50:50 (v/v) methanol:water for 15 min, followed by centrifugation and direct injection of the supernatant. Quantification was performed using external calibration with commercially available standards of vitexin (Cayman Chemical, Ann Arbor, USA) and isovitexin (HWI Group, Rülzheim, Germany). The calibration range covered typical phytopharmaceutical levels, with concentrations between approximately 0.3 and 10 mg/g for both analytes across the investigated samples.

### Spectroscopic measurements

For all spectroscopic measurements, samples were analyzed in the dry state. Approximately 2 g of the powdered material was transferred into quartz sample cups (diameter 34 mm) with a flat optical window for spectra measurements. For intact samples, whole material was directly placed into the same cups, with complete coverage of the measurement window. All spectra were acquired in diffuse reflectance mode at room temperature, with rotation or repositioning of the sample between replicate scans to minimize local heterogeneity effects. In each case, triplicate measurements were acquired for all samples and subsequently averaged prior to multivariate analysis, in accordance with standard procedures aiming to minimize short-term spectral variability.

Measurements were performed using four NIR spectrometers differing in size, design, and analytical capabilities. The Büchi NIRFlex N-500 (benchtop; Büchi AG, Flawil, Switzerland) served as the high-performance reference system. This Fourier transform (FT)-NIR instrument is uniquely equipped with a polarization interferometer (TeO₂ wedge design) and a thermoelectrically cooled extended-range InGaAs detector. Spectra were acquired in diffuse reflectance mode using the solid sample module over a spectral range of 10,000–4000 cm⁻^1^ at a resolution of 8 cm⁻^1^, with 64 scans averaged per measurement. The N-500 offers high spectral resolution, excellent signal-to-noise ratio, and long-term stability under laboratory conditions.

Three miniaturized, handheld devices were applied for comparison. The MicroNIR 2200 (Viavi Solutions, San Jose, USA) covers the range 8000–4651 cm⁻^1^ using a linear variable filter (LVF) combined with an InGaAs array detector with 128 pixels. Spectra were collected using manufacturer-recommended settings; in the case of MicroNIR 2200, with 200 scans averaged and a 6.5-ms acquisition window per each scan. The MicroNIR 1700 ES (Viavi Solutions, San Jose, USA) is a newer model in the same product line, covering 10,526–6060 cm⁻^1^ (125 wavelengths are recorded by the 128-element array detector; nominal resolution of 12.5 nm at 1000 nm and 25 nm at 2000 nm); it offers a higher signal-to-noise ratio, and is equipped with improved temperature compensation for extended operational stability in field applications. Spectra were collected using the recommended settings: 1000 averaged scans, with a 7.5-ms acquisition window per each scan. While both MicroNIR models use LVF-based wavelength selection and are technologically similar, their subtle differences in wavelength range and instrument stability are relevant for the present study. The multi-channel MicroNIR instruments represent a very high degree of miniaturization and a flexible application as remote sensors limited only by the USB interface for power delivery and control/data transfer.

On the other hand, the Thermo Scientific microPHAZIR (Thermo Fisher Scientific Inc., Waltham, USA) was employed as a fully self-contained (autonomous) handheld instrument based on a MEMS-based scanning interferometer (a resonantly driven micromirror, suspended on springs and actuated electrostatically), a single-element InGaAs detector, battery power, and an LCD display. The instrument is characterized by good spectral resolution and stability; however, relative to other instruments, it operates over a very narrow wavelength window. Nonetheless, it covers the low-frequency part of the NIR region, rich in analytical information from numerous binary combination bands [37, 38]. The spectra collected using microPHAZIR were processed in the wavenumber range of 6250–4170 cm⁻^1^ at a nominal resolution of 11 nm (~16 cm^−1^), using 50 averaged scans per measurement.

### Data pretreatments and multivariate modelling

NIR spectra were subjected to a standardized pretreatment sequence prior to multivariate modelling, designed to progressively reduce baseline shifts, multiplicative scatter effects, and residual background variance unrelated to the analytes. The applied sequence consisted of Savitzky-Golay (SG) second derivative transformation (polynomial order 2, window size 9 [4+1+4] points) to enhance spectral detail and compensate for baseline variability, followed by standard normal variate (SNV) correction to mitigate multiplicative scattering and scaling differences between samples. In this study, the pretreatment sequence was developed systematically. The results showed high stability; the SG-2–9 + SNV pretreatment was often optimal (Electronic Supplementary Material, Figs. S1–S2). The pretreatment vs. per-instrument best settings yielded only modest differences and do not change the qualitative LOD/LOQ relationships discussed in the paper.

Consequently, the pretreatment scheme was deliberately fixed across instruments to improve clarity and emphasize the core objective, the evaluation of LOD/LOQ frameworks rather than device-specific tuning. This approach maintains comparability under a PAT-style standardized workflow. The selected scheme (SG 2nd derivative, 9-point window, polynomial order 2, followed by SNV) was verified to lie within the flat optimum region for all instruments and sample types (see Electronic Supplementary Material, Figs. S1–S2); minor parameter variations (e.g., derivative order or window width) produced only marginal RMSECV differences and did not affect the LOD/LOQ relationships. We should note, for microstructured plant matrices (intact or homogenized) in diffuse reflectance NIR, a first-order scatter correction (SNV or, equivalently, MSC) is standard practice and, in practical terms, almost mandatory to suppress baseline offsets and multiplicative pathlength effects; the preceding or following steps (derivatives, OSC) can address wavelength-dependent or orthogonal variance. In the present case, MSC delivered comparable results; SNV was preferred because it is reference-free and parameter-free, reduces additive/multiplicative scatter without relying on a representative reference spectrum.

OSC was further applied in selected models to enhance analyte signal delineation within the LV space by removing variance orthogonal to the reference concentrations. OSC was implemented with a single orthogonal component and was applied only after derivative and SNV processing. Models both with and without OSC were developed to evaluate its specific impact on model performance and detection capability estimation. Given the distinct ways in which variance-leverage mLOD and NAS-LOD relate to model internal geometry, the structural reorganizations induced by OSC are particularly of interest in the present study.

All multivariate calibration models were developed using PLS-R, applied independently for each spectrometer and target analyte. Separate PLS-R models were built for vitexin, isovitexin, and their sum concentration (vitexin and isovitexin), depending on the analytical objective. All models were constructed using mean-centered data following the spectral pretreatment sequence described above. The optimal number of LVs was selected using cross-validation (CV), based on the model configuration minimizing the root mean square error of cross-validation (RMSECV) while avoiding overfitting. Leave-one-out cross-validation (LOOCV) was implemented using 50 segments, with each segment representing one averaged sample (triplicate spectra averaged prior to modelling) to deliver independent error estimation across the full dataset. In each case, the model validity was independently confirmed via test set validation (TSV) on an external test set (*n* = 10). Model development and validation were performed using The Unscrambler X (version 11, CAMO Software, Oslo, Norway), with supplementary analyses and LOD/LOQ calculations conducted in MATLAB (R2020b, The MathWorks Inc., Natick, USA).

### LOD and LOQ estimation

Detection and quantification limits (LOD/LOQ) for each PLS-R model were estimated using two conceptually distinct multivariate frameworks: the variance-driven, sensitivity-ratio-based approach following Allegrini and Olivieri (mLOD/mLOQ) [23], and the net analyte signal–based approach (NAS-LOD/NAS-LOQ) introduced by Lorber [24, 25].

The former mLOD/mLOQ framework follows the IUPAC-compliant procedure, which estimates the LOD based on instrumental variance, regression vector norm, and the statistical leverage of a blank sample (or lowest concentration sample if the blank is not feasible) within the score space [23]. LOD values were derived using a geometric formulation that combines average spectral variance (var(**X**)), calibration variance (var(**y**)), and score-based leverage terms. This approach avoids direct use of residual prediction errors (e.g., RMSECV or RMSEP) and instead reflects a structural sensitivity concept consistent with IUPAC’s multivariate detection framework, which describes the inherent detectability of an analyte based on the spatial configuration and variability of the model’s internal structure (latent space of the model). This framework represents a geometric, variance-weighted estimate of how well-separated the blank is from a detectable signal level in the PLS latent space, considering the typical variation and leverage of the calibration set (Eq. 1, and for clarity of presentation will be named variance-based mLOD/mLOQ in this work.

1 $$\mathrm{mLOD}= 3.3\,\sqrt{ \frac{\sigma_x^{2}\,(1+h_{0})}{\left\lVert \mathbf{b} \right\rVert^{2}} \;+\; h_{0}\,\sigma_y^{2}}$$

where ${\sigma }_{x}^{2}$ is the mean instrumental variance across wavelengths (var(**X**)), *h*_0_ is the leverage of the blank, ${\sigma }_{y}^{2}$ is the variance of the calibration *y*-values (var(**y**)), and **b** is the PLS regression vector. Subsequently, mLOQ values were derived directly from the mLOD (Eq. 2), using a fixed proportionality factor of 3.33 (i.e., LOQ ≈ 10*σ* vs. LOD ≈ 3*σ*), consistent with IUPAC rationale.

2 $$\mathrm{mLOQ}= \frac{10}{3.3}\,\mathrm{mLOD}\;\approx\; 3.03\,\mathrm{mLOD}$$

Accordingly, the minimum (mLOD_min_ and mLOQ_min_) values were obtained using the leverage of the lowest concentration sample (*h*_0_), while the maximum (mLOD_max_ and mLOQ_max_) values were obtained for the maximum leverage across the calibration set (${h}_{0}^{\text{max}}$) to account for worst-case alignment. Note, replacing a true blank (unavailable in phytopharmaceuticals) with the lowest concentration sample makes the mLOD estimate more conservative (i.e., estimated LOD values being more pessimistic), which is generally appropriate and even desirable in real-world PAT applications.

The NAS-based LOD/LOQ approach was calculated from the magnitude of the analyte-specific NAS vector, which represents the portion of the regression vector uniquely aligned with the analyte signal, after orthogonalization against all other LVs. Accordingly, the NAS vector ${\text{w}}_{\text{NAS}}$ (Eq. 3) was derived from the selected (associated with LV1) weight vector **w**, with its projections onto the remaining **X**-loadings space removed:

3 $$\mathbf{w}_{\mathrm{NAS}}= \mathbf{w} - \mathbf{P}\bigl(\mathbf{P}^{\top}\mathbf{w}\bigr)$$

where **P** is the matrix of **X**-loadings excluding the analyte-representing LV. The resulting Euclidean norm ∥**w**_NAS_∥ quantifies the strength of the analyte-specific component, and was used in NAS-LOD and NAS-LOQ estimation as described by Eqs. 4 and 5.

4 $$\mathrm{NAS}\text{-}\mathrm{LOD}= \frac{3\,s_{a}}{\left\lVert \mathbf{w}_{\mathrm{NAS}} \right\rVert }$$

5 $$\mathrm{NAS}\text{-}\mathrm{LOQ}= \frac{10\,s_{a}}{\left\lVert \mathbf{w}_{\mathrm{NAS}} \right\rVert }$$

In Eqs. 4 and 5, *s*_a_ is the residual standard deviation of prediction estimated via CV. NAS was calculated using LV1 to standardize the approach and evaluate the impact of OSC on the modelling procedure. Using LV1 makes NAS-LOD conservative if analyte information is spread over higher LVs; we accept this to avoid subspace selection and rotation ambiguity. The number of retained LVs still influences NAS-LOD via the cross-validated residual variance, which generally decreases as LVs are added up to the CV-optimal model.

Calculations were implemented in MATLAB (R2020b) using custom scripts. Both LOD frameworks were evaluated for models with and without OSC pretreatment, allowing for a systematic comparison of their behaviour. All LOD/LOQ values were consistently calculated in mg/g units. The impact of the number of LVs on LOD/LOQ estimates was further investigated through model truncation, particularly for selected models of higher complexity.

## Results and discussion

### NIR spectra

**Fig. 1:**
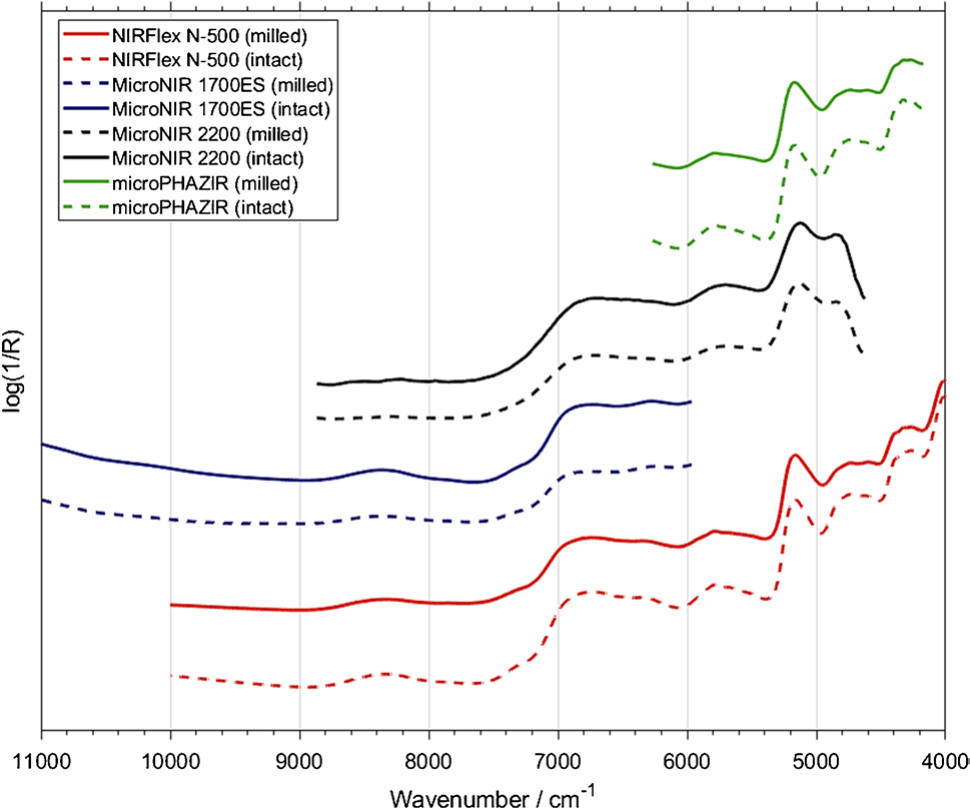
NIR spectra presented for one representative sample of *Passiflora incarnata* (intact and milled form) measured using all the spectrometers included in the study. Spectra presented with an arbitrary uniform shift on the intensity axis (vertical offset) for clarity of presentation

NIR spectra of a representative intact sample measured across all spectrometers are presented in Fig. 1. Despite differences in spectral resolution, wavelength coverage, and optical design, all instruments captured the major absorption features characteristic of phytopharmaceutical matrices. These include broad overtone and combination bands associated with O–H, C–H, and N–H functional groups, typically arising from moisture, carbohydrates, proteins, and other plant constituents. Nonetheless, some distinctions between instruments were observable in the measured lineshape characteristics. Using the benchtop N-500 (with its high stability FT-NIR configuration (section Spectroscopic Measurements)) as the reference, certain contrasting features apparent in the spectra measured by portable LVF-based instruments (the MicroNIR 2200 and 1700 ES) are noticeable. Both these sensors displayed narrower coverage and mild baseline fluctuations, while the 2200 model also featured a rapid decrease in the measured intensity towards the low-frequency boundary (Fig. 1). Yet, even the MicroNIR 2200 retained sufficient spectral fidelity and consistency in critical regions for reliable model construction. On the other hand, the microPHAZIR also reproduced the spectral lineshape of the sample in a specific manner, while featuring the narrowest usable spectral range (6250–4170 cm⁻^1^) as well, limited to combination band regions. Still, the spectrometer captured informative matrix- and moisture-related features relevant for reliable calibration.

### PLS regression models

**Fig. 2:**
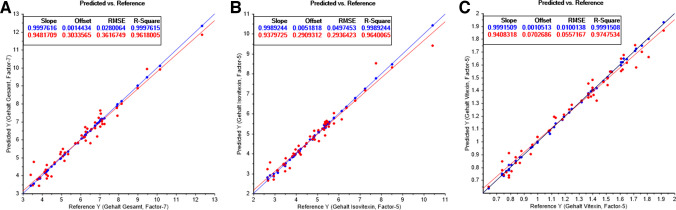
PLS regression line obtained from Büchi NIRFlex N-500 for **A** vitexin + isovitexin, **B** isovitexin, **C** vitexin

The key performance parameters of all developed PLS-R models, constructed separately for each spectrometer, analyte (vitexin, isovitexin, total content), and sample condition (milled/intact), are summarized in Tables 1, 2 and 3. The modelling strategy and evaluation metrics applied are described in the section. As expected, the Büchi NIRFlex N-500 (benchtop FT-NIR) consistently delivered the highest model performance across all analytes and conditions. For milled samples, quantification of vitexin and isovitexin content yielded excellent results, with calibration coefficients of determination (*R*^2^_C_) exceeding 0.99 and prediction errors (RMSEP) typically below 0.5 mg/g and, in the case of vitexin quantification, even below 0.1 mg/g (Tables 1, 2 and 3). These results reflect the upper performance benchmark achievable under controlled laboratory conditions using high-end benchtop instrumentation. The model fit quality for the reference benchtop spectrometer, on the example of intact samples is presented in Fig. 2A–C, for the combined quantification of both analytes (vitexin and isovitexin), and individual ones, accordingly.

In contrast, predictive performance declined when using portable or handheld devices, reflecting inherent instrumental limitations such as reduced spectral resolution, narrower wavelength ranges, and increased noise levels. This trend was most pronounced for the microPHAZIR, which combines narrow spectral coverage with single-channel detection. Additionally, sample condition had a major impact on model quality across all spectrometers. Milled samples consistently produced better predictive results compared to intact material, due to reduced heterogeneity and lower scattering variability, which directly affect NIR spectra and model stability.

**Table 1:** PLS-R performance metrics for simultaneous quantification of vitexin and isovitexin

Spectrometer	Sample	*R*^2^_C_	RMSEC (mg/g)	*R*^2^_CV_	RMSECV (mg/g)	*R*^2^_TSV_	RMSEP (mg/g)	LVs
N-500	Milled	0.994	0.14	0.979	0.27	0.961	0.22	6
N-500	Intact	0.999	0.03	0.962	0.36	0.899	0.42	7
MicroNIR 2200	Milled	0.947	0.42	0.930	0.49	0.909	0.32	2
MicroNIR 2200	Intact	0.995	0.13	0.959	0.38	0.884	0.39	2
microPHAZIR	Milled	0.933	0.47	0.892	0.61	0.828	0.51	2
microPHAZIR	Intact	0.805	0.80	0.744	0.94	0.588	0.69	2
MicroNIR 1700 ES	Milled	0.932	0.47	0.920	0.52	0.768	0.64	3
MicroNIR 1700 ES	Intact	0.842	0.72	0.832	0.76	0.847	0.67	2

**Table 2:** PLS-R performance metrics for quantification of isovitexin

Spectrometer	Sample	*R*^2^_C_	RMSEC (mg/g)	*R*^2^_CV_	RMSECV (mg/g)	*R*^2^_TSV_	RMSEP (mg/g)	LVs
N-500	Milled	0.993	0.13	0.977	0.24	0.832	0.31	4
N-500	Intact	0.999	0.05	0.964	0.29	0.920	0.26	9
MicroNIR 2200	Milled	0.947	0.35	0.924	0.43	0.594	0.46	3
MicroNIR 2200	Intact	0.997	0.08	0.974	0.25	0.760	0.45	2
microPHAZIR	Milled	0.924	0.42	0.908	0.47	0.759	0.44	2
microPHAZIR	Intact	0.806	0.67	0.751	0.77	0.642	0.56	2
MicroNIR 1700 ES	Milled	0.934	0.39	0.921	0.44	0.811	0.47	2
MicroNIR 1700 ES	Intact	0.845	0.60	0.833	0.63	0.540	0.66	2

**Table 3:** PLS-R performance metrics for quantification of vitexin

Spectrometer	Sample	*R*^2^_C_	RMSEC (mg/g)	*R*^2^_CV_	RMSECV (mg/g)	*R*^2^_TSV_	RMSEP (mg/g)	LVs
N-500	Milled	0.998	0.01	0.971	0.06	0.907	0.09	4
N-500	Intact	0.999	0.01	0.975	0.06	0.914	0.10	3
MicroNIR 2200	Milled	0.925	0.09	0.915	0.10	0.842	0.09	2
MicroNIR 2200	Intact	0.995	0.03	0.952	0.08	0.708	0.11	3
microPHAZIR	Milled	0.890	0.11	0.886	0.12	0.851	0.11	2
microPHAZIR	Intact	0.755	0.17	0.743	0.18	0.719	0.18	1
MicroNIR 1700 ES	Milled	0.919	0.10	0.872	0.13	0.906	0.10	2
MicroNIR 1700 ES	Intact	0.907	0.10	0.842	0.14	0.720	0.13	2

Sample state further contributed to performance variability; models based on milled samples typically outperformed models based on intact samples, which is a common trend reported in similar studies [39, 40]. This behaviour is attributed to the reduced scattering variability and increased homogeneity achieved by particle size reduction, which suppresses multiplicative and baseline effects that otherwise obscure chemically relevant information in intact samples.

The microPHAZIR, representing the narrowest spectral coverage among the tested instruments, produced the lowest overall model performance. In several cases, test set coefficients of determination (*R*^2^_TSV_) fell below 0.85, and prediction errors approached 0.5 mg/g. This suggests that the quantitative performance was predominantly limited by intrinsic spectral features of the low-frequency NIR range. However, even under these constrained conditions, the resulting models remained quantitatively meaningful, with absolute errors well within the typical good results for quantitative analysis of phytopharmaceutical marker compounds (0.3–10 mg/g). This confirms the suitability of practical in-field deployment of miniaturized NIR instrumentation even in demanding analytical contexts.

Importantly, while Tables 1, 2 and 3 demonstrate the variations in model performance across spectrometers and sample types, they do not directly reveal the actual detection capability of the models in the sense of quantitative LOD or LOQ. Furthermore, these metrics can mask poor analyte alignment, diverging from the PAT principle of structure-transparent and interpretable analytical method. As detailed in the section, the ability of a model to detect and quantify low analyte levels is governed not only by its overall predictive error but by how the analyte-specific variance is represented and isolated within the latent space. This distinction becomes critical when evaluating LOD/LOQ behaviour under different theoretical frameworks.

### Comparison of variance-leverage mLOD and NAS-LOD estimates

While the calculation of LOD/LOQ represents a fundamental element of quantitative analytical chemistry, its application within multivariate calibration, particularly in phytopharmaceutical products, remains challenging. Tables 4, 5 and 6 systematically compare the two conceptually distinct frameworks for multivariate LOD/LOQ estimation: (i) the variance-leverage mLOD framework and (ii) the NAS-based approach, focusing on the magnitude and delineation of the analyte-specific signal within the latent structure of the PLS-R model.

Both LOD/LOQ estimation frameworks were evaluated using the optimized PLS-R models for vitexin, isovitexin, and their sum (Tables 4, 5 and 6), as quantified across different spectrometers and sample conditions. The modelling was performed using real phytopharmaceutical samples of *Passiflora incarnata* L., characterized by substantial matrix variability arising from diverse cultivation origins, processing conditions, and inherent physicochemical heterogeneity. The impact of these sample properties on multivariate LOD/LOQ estimation was systematically investigated. A key observation emerging from this study was the divergent behaviour of variance-leverage mLOD and NAS-LOD estimates with increasing model complexity, i.e., with the inclusion of additional LVs. As anticipated, the variance-leverage mLOD values generally improved (decreased) in models with fewer LVs. This reflects the favorable model geometry and reduced calibration variance associated with more compact latent structures, causing reduced average leverage and better separation between the blank and analyte signal within the latent space. Conversely, NAS-LOD values decreased with increasing numbers of LVs, reflecting their direct dependence on the concentration and geometric delineation of analyte-related variance within the latent space. Given that NAS is computed on LV1, the decrease of NAS-LOD with increasing LVs is driven by the drop in CV residual variance. When analyte signal is dispersed across LVs (notably without OSC), NAS_LV1_ is conservative by design, whereas OSC tends to concentrate the analyte in LV1, making NAS_LV1_ both more interpretable and less conservative.

**Table 4:** Multivariate LOD/LOQ estimates (in mg/g) for vitexin and isovitexin (sum): variance-leverage mLOD and NAS-LOD

Spectrometer	Sample	mLOD_min_	mLOD_max_	mLOD_avg_	mLOQ_min_	mLOQ_max_	mLOQ_avg_	NAS-LOD	NAS-LOQ	LVs
N-500	Milled	3.22	5.11	4.17	9.77	15.48	12.62	2.07	6.91	6
N-500	Intact	3.25	4.91	4.08	9.85	14.88	12.36	2.04	6.79	7
MicroNIR 2200	Milled	1.06	2.86	1.96	3.21	8.68	5.94	1.76	5.87	2
MicroNIR 2200	Intact	1.82	3.73	2.77	5.51	11.30	8.40	3.17	10.56	2
microPHAZIR	Milled	1.27	3.04	2.16	3.84	9.22	6.53	3.42	11.40	2
microPHAZIR	Intact	1.33	2.81	2.07	4.04	8.52	6.28	11.37	37.88	2
MicroNIR 1700 ES	Milled	1.98	4.04	3.01	5.99	12.23	9.11	3.36	11.19	3
MicroNIR 1700 ES	Intact	1.31	3.30	2.31	3.96	10.01	6.98	4.25	14.16	2

**Table 5:** Multivariate LOD/LOQ estimates (in mg/g) for isovitexin: variance-leverage mLOD and NAS-LOD

Spectrometer	Sample	mLOD_min_	mLOD_max_	mLOD_avg_	mLOQ_min_	mLOQ_max_	mLOQ_avg_	NAS-LOD	NAS-LOQ	LVs
N-500	Milled	2.40	4.05	3.22	7.26	12.27	9.76	4.85	16.16	4
N-500	Intact	2.67	4.83	3.75	8.08	14.63	11.35	0.45	1.50	9
MicroNIR 2200	Milled	0.97	3.03	2.00	2.93	9.19	6.06	1.27	4.24	3
MicroNIR 2200	Intact	1.68	3.29	2.49	5.10	9.96	7.53	2.32	7.74	2
microPHAZIR	Milled	1.14	2.76	1.95	3.46	8.37	5.92	2.81	9.36	2
microPHAZIR	Intact	0.71	2.50	1.60	2.14	7.58	4.86	9.24	30.79	2
MicroNIR 1700 ES	Milled	1.08	2.83	1.95	3.26	8.56	5.91	3.08	10.25	2
MicroNIR 1700 ES	Intact	1.22	3.02	2.12	3.70	9.14	6.42	3.24	10.81	2

**Table 6:** Multivariate LOD/LOQ estimates (in mg/g) for vitexin: variance-leverage mLOD and NAS-LOD

Spectrometer	Sample	mLOD_min_	mLOD_max_	mLOD_avg_	mLOQ_min_	mLOQ_max_	mLOQ_avg_	NAS-LOD	NAS-LOQ	LVs
N-500	Milled	0.66	0.78	0.72	1.99	2.37	2.18	1.37	4.56	4
N-500	Intact	0.60	0.65	0.62	1.82	1.96	1.89	2.94	9.81	3
MicroNIR 2200	Milled	0.31	0.39	0.35	0.93	1.19	1.06	0.59	1.96	2
MicroNIR 2200	Intact	0.31	0.51	0.41	0.93	1.54	1.24	0.86	2.88	3
microPHAZIR	Milled	0.42	0.46	0.44	1.28	1.40	1.34	0.82	2.72	2
microPHAZIR	Intact	0.58	0.64	0.61	1.75	1.94	1.85	2.41	8.03	1
MicroNIR 1700 ES	Milled	0.34	0.44	0.39	1.04	1.35	1.19	1.35	4.48	2
MicroNIR 1700 ES	Intact	0.30	0.50	0.40	0.90	1.50	1.20	1.22	4.08	2

This opposite behaviour was consistently observed across spectrometers and analytes, and represents a fundamental consequence of the distinct mechanisms underlying both frameworks. The observed divergence in LOD trends (Tables 4, 5 and 6) very well reflects the difference between the global sensitivity measured by mLOD and the analyte-specific isolation measured by NAS-LOD. The results demonstrate that neither framework should be regarded as universally superior or universally applicable. In matrices with dominant or highly variable background contributions—such as phytopharmaceutical extracts—variance-leverage mLOD may underestimate detection capability if analyte variance is weakly expressed relative to total model variance. Conversely, although not observed in the present results, NAS-LOD values may exhibit overly optimistic behaviour in highly flexible or over-parameterized models, given that a higher number of retained LVs tends to decrease the LOD estimate. This interplay underscores the critical role of model structure and interpretability in multivariate LOD/LOQ estimation. The dependence of NAS-LOD on the geometric separation of analyte-relevant variance is both its advantage (in transparent, well-structured models) and its potential limitation (in unstable or poorly aligned latent spaces). Conversely, the variance-leverage mLOD provides a more global performance estimate, inherently robust to LV rotations, but less sensitive to the true representation of analyte variance within the latent structure.

Particular attention should be given to the limits and interpretative role of multivariate LOD/LOQ conventions. While both frameworks offer valuable perspectives for assessing detection capability in multivariate calibration, their applicability in highly variable phytopharmaceutical systems carries inherent limitations. In complex matrices, increased model flexibility (arising from a higher number of LVs) or elevated calibration variance may lead to reduced separation between low-concentration samples and the calibration cloud in the latent space, resulting in unstable or overly optimistic mLOD estimates that underestimate the actual detection challenges. In contrast, NAS-LOD is directly dependent on the geometric isolation of analyte variance within the model. In cases where the analyte signal is weak, fragmented, or distributed across several LVs—typical for samples with high matrix interference—the resulting NAS vector may become small, yielding conservative, potentially excessively pessimistic LOD estimates, despite adequate external prediction performance.

### Behaviour and diagnostic role of LOD/LOQ frameworks in phytopharmaceutical PAT calibration

The behaviour of LOD and LOQ estimates in PLS-R is fundamentally driven by the internal latent structure of the calibration model, yet the nature and extent of this dependency differ considerably between the variance-based mLOD and NAS-LOD frameworks, as outlined in the section. Two complementary diagnostics allowed systematic quantification of latent structure quality in relation to these LOD frameworks: (i) the variance of the reference analyte explained by each LV, internally evaluating analyte signal compactness or dispersion within the model, and (ii) projection of the pure analyte spectrum onto the model LVs, serving as an external estimate of analyte representation within the latent structure.

Across the considered cases, substantial differences in estimated LOD/LOQ were observed depending on the framework (NAS-LOD vs. variance-based mLOD), model complexity, and preprocessing strategy. Using the N-500 for vitexin and isovitexin quantification, NAS-LOD values below 1 mg/g were achieved consistently in milled samples, slightly increasing in intact samples (Tables 4, 5 and 6). Portable spectrometers, particularly the MicroNIR 2200 and 1700 ES, produced NAS-LOD values generally ranging between 1.5 and 4 mg/g, occasionally exceeding 5 mg/g in intact samples without OSC. Variance-based mLOD estimates exhibited less numerical variability and were less responsive to differences in sample state or preprocessing conditions. Nevertheless, absolute NAS-LOD and mLOD values often converged in well-structured models, suggesting alignment under conditions of optimal analyte delineation. In the case of the microPHAZIR, the LOD values determined through both frameworks led to consistently higher values, particularly for intact samples (Tables 4, 5 and 6), reflecting the intrinsic limitations of spectral range and lower analyte alignment within the latent space.

#### LV dependence of NAS-LOD versus variance-based mLOD

A key observation was the divergent behaviour of variance-leverage mLOD and NAS-LOD as the number of retained LVs increased. The values of mLOD improved for more compact models (lower number of LVs), while NAS-LOD computed on LV1 decreased as additional LVs were retained, due to the reduction of the CV residual variance term (the NAS direction remained LV1). For a directly comparable example under identical conditions (same instrument, analyte, sample state, and OSC setting), the N-500, vitexin, intact, non-OSC model shows NAS-LOD decreasing from 13.13 mg g⁻^1^ (*F* = 3) to 8.65 mg g⁻^1^ (*F* = 5), while mLOD_avg_ increases slightly from 3.45 to 3.58 mg g⁻^1^ (Table 7; N-500, V/i, without OSC). In the corresponding OSC-pretreated model (N-500, vitexin, intact, OSC), NAS-LOD further decreases from 2.94 mg g⁻^1^ (*F* = 3) to 1.13 mg g⁻^1^ (*F* = 5), accompanied by a minor change in mLOD_avg_ from 0.62 to 0.70 mg g⁻^1^ (Table 7; N-500, V/i, OSC). Together, these controlled examples clearly illustrate the opposite dependence of the two LOD frameworks on model complexity under otherwise identical conditions.

This inverse LV dependence highlights the different roles of the two frameworks. NAS-LOD_LV1_ quantifies the analyte-aligned signal-to-residual contrast in X-space, while the variance-leverage mLOD reflects global predictive sensitivity and leverage-dependent prediction uncertainty. Considering both the analyte-specific delineation (NAS-LOD) and the global detectability conditioned on leverage (mLOD) provides a complementary and more complete model assessment.

#### Influence of OSC preprocessing on latent structure and LOD

OSC pretreatment strongly and systematically influenced LOD estimates, predominantly through direct modification of the internal latent structure. The most substantial impact was observed in NAS-LOD values, particularly in intact samples and portable instruments, where background interference was most prominent. OSC-pretreated models consistently achieved meaningful NAS-LOD reductions (Table 7), a direct result of improved analyte signal concentration and isolation within early LVs. The table presents exemplary results for the benchtop and miniaturized spectrometer and varying model complexity (retained LVs) in each case, to assess the stability in the behaviour and potential trends that LOD/LOQ frameworks may follow in a range of method parameters.

**Table 7:** LOD and LOQ estimates (in mg/g) using variance-based mLOD and NAS-LOD conventions with and without OSC included in the pretreatment schemes. Analysis type: vitexin (V), isovitexin (Iso), intact (i)

Spectr.	Anal.	OSC	*R*^2^ (CV)	RMSECV (mg/g)	mLODmin	mLODmax	mLOD avg	mLOQ min	mLOQ max	mLOQ avg	NAS-LOD	NAS-LOQ	*F*
N-500	V/i	-	0.639	0.21	3.36	3.53	3.45	10.19	10.71	10.45	13.13	43.75	3
N-500	V/i	-	0.616	0.22	3.48	3.67	3.58	10.54	11.13	10.84	8.65	28.85	5
N-500	V/i	Yes	0.952	0.08	0.60	0.65	0.62	1.82	1.96	1.89	2.94	9.81	3
N-500	V/i	Yes	0.975	0.06	0.62	0.78	0.70	1.89	2.37	2.13	1.13	3.76	5
1700 ES	Iso/i	-	0.632	0.94	1.88	3.17	2.52	5.71	9.59	7.65	7.56	25.19	4
1700 ES	Iso/i	Yes	0.833	0.63	1.22	3.02	2.12	3.70	9.14	6.42	3.24	10.81	2

As anticipated for phytopharmaceutical matrices, the inclusion of OSC in the pretreatment scheme has a significant impact on the predictive power of the models, monitored via routine parameters (*R*^2^, RMSE). However, multivariate LOD/LOQ values add considerable depth to the diagnostic layer that provides refined control over the method. A moderate improvement was observed in the isovitexin model using MicroNIR 1700 ES, where mLODavg decreased from 2.52 mg/g (non-OSC) to 2.12 mg/g (OSC). Variance-based mLOD also showed substantial sensitivity to OSC pretreatment in the vitexin model (N-500, intact), where mLODavg decreased significantly from 3.45–3.58 mg/g (non-OSC) to 0.62–0.70 mg/g (OSC), depending on the number of LVs. In this case, OSC reduced mLODavg by factors of approximately 5.6 (3 LVs) and 5.1 (5 LVs), while NAS-LOD dropped by approximately 4.5 and 7.7 times, respectively. These NAS-LOD improvements are consistent with the anticipated higher sensitivity to analyte signal concentration and orthogonality of this multivariate LOD framework.

Additionally, leverage-driven uncertainty narrowed after OSC, as indicated by smaller mLOD min–max intervals (e.g., N-500, vitexin, intact: from 0.17–0.19 mg g⁻^1^ pre-OSC to 0.05–0.16 mg g⁻^1^ post-OSC). As the number of retained LVs increased, we observed the characteristic inverse trend; mLOD_avg_ rose slightly, whereas NAS-LOD decreased. For the same controlled case (N-500, vitexin, intact, non-OSC), mLOD_avg_ increased from 3.45 to 3.58 mg g⁻^1^ as the number of LVs increased from 3 to 5, while NAS-LOD fell from 13.13 to 8.65 mg g⁻^1^ (Table 7; N-500, V/i, without OSC). In the corresponding OSC model, NAS-LOD decreased from 2.94 to 1.13 mg g⁻^1^ (*F* = 3–5), with mLOD_avg_ changing from 0.62 to 0.70 mg g⁻^1^ (Table 7; N-500, V/i, OSC). These trends highlight the greater structural responsiveness of NAS-LOD to latent space reorganization, while mLOD remained more conservative and less affected by analyte signal fragmentation in the model structure.

**Fig. 3:**
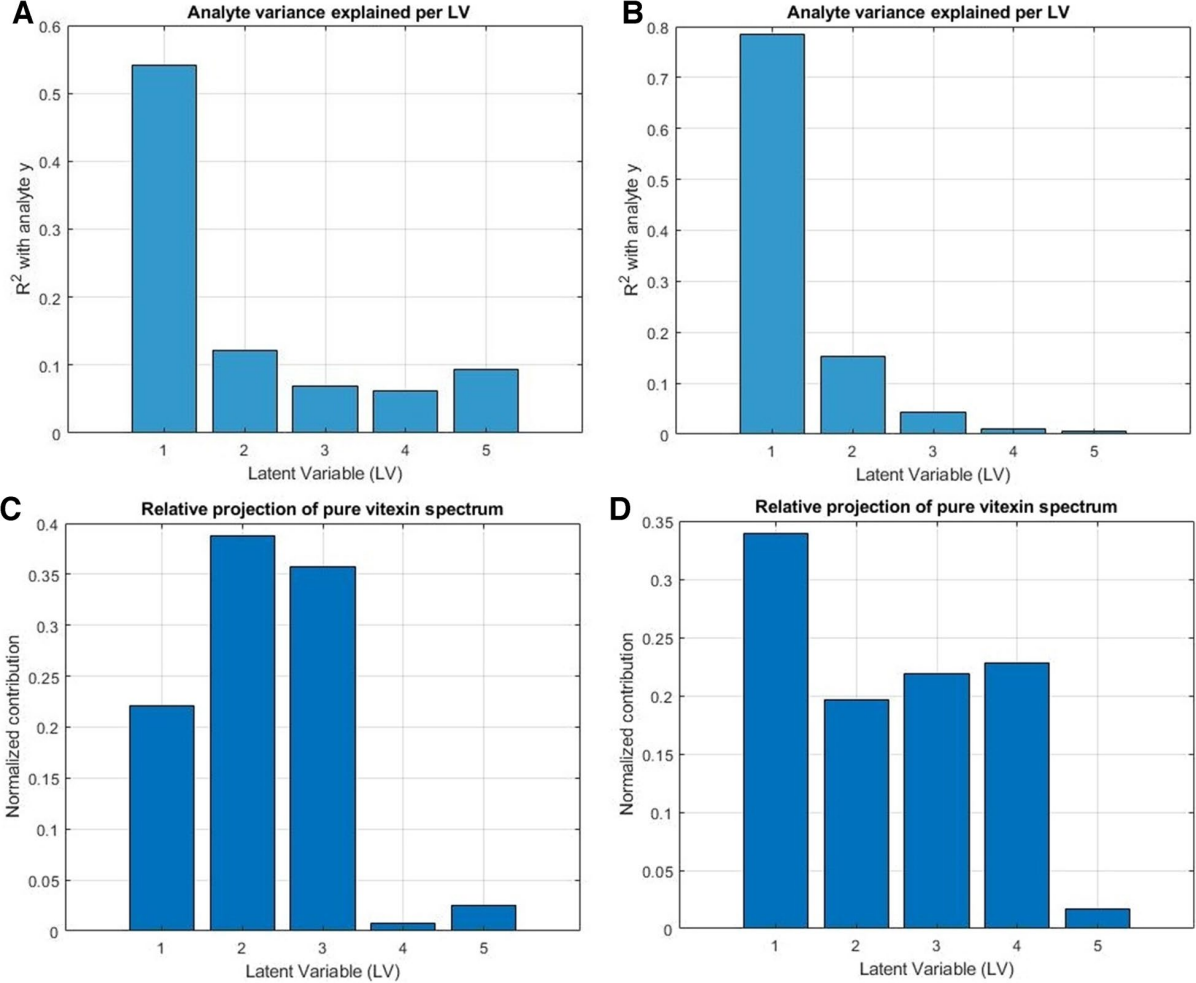
Comparison of PLS-R structure without OSC (**A**, **C**) and with OSC (**B**, **D**) included in the pretreatment scheme preceding modelling of vitexin (N-500, intact samples). **A**, **B** Explained variance of reference analyte per LV, illustrating analyte signal concentration in OSC model (dominance of LV1) versus dispersion across multiple LVs in the non-OSC model. **C**, **D** Projection of the pure vitexin standard spectrum onto the latent space (**X**-loadings), indicating stronger alignment with early LVs in the OSC model

**Fig. 4:**
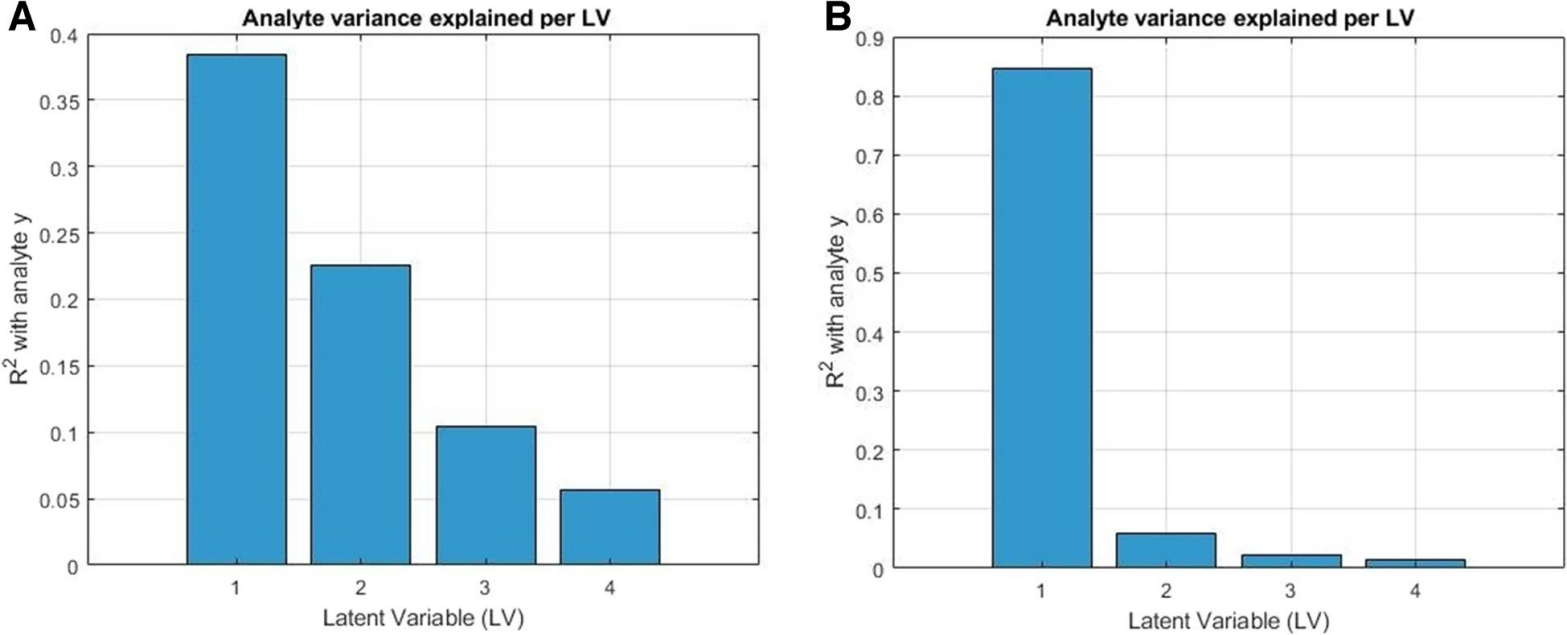
Comparison of PLS-R structure (presented for 4 LVs) without OSC (**A**) and with OSC (**B**) included in the pretreatment scheme preceding modelling of isovitexin (MicroNIR 1700 ES, intact samples). **A**, **B** Explained variance of reference analyte per LV, illustrating analyte signal concentration in OSC model (dominance of LV1) versus dispersion across multiple LVs in the non-OSC model

Internal diagnostic analysis supported these observations, revealing a marked increase in the proportion of analyte variance captured by LV1 and reduced contributions from higher-order LVs after the application of OSC. This structural shift induced by OSC was quantitatively confirmed by latent variable diagnostics. For the sake of comparison, two examples are provided selected by how the PLS-R latent structure and LOD/LOQ values respond to orthogonal correction. The example selected for the benchtop spectrometer showed a less profound shift, which is also reflected by comparable optimal model complexity (i.e., number of retained LVs) in both OSC and non-OSC scenarios. In contrast, the second example, drawn from models developed using a miniaturized spectrometer, demonstrated a much more pronounced structural reorganization, which substantially altered the optimal model complexity. Accordingly, as shown in Fig. 3A, B, the proportion of analyte variance (vitexin, N-500) explained by LV1 increased markedly in the OSC-pretreated model, while variance in higher LVs diminished. External projection of the pure vitexin spectrum onto the model LVs (Fig. 3C, D) further confirmed this alignment, with the OSC model showing stronger and more focused projection onto LV1. A similar behaviour was observed for isovitexin models based on the MicroNIR 1700 ES (Fig. 4). The analyte variance, originally spread across multiple LVs in the uncorrected model, became sharply concentrated in LV1 (*R*^2^ of 0.85) once OSC is included in the pretreatment scheme. This results in a stronger analyte alignment and thus more transparent model structures in the pretreated datasets. Note that the projection was based on the spectrum of a vitexin reference standard, pretreated in a way consistent with the spectra of the natural samples. While such a spectrum — recorded on a pure, polycrystalline substance — cannot fully replicate the analyte signal as it exists within the complex phytopharmaceutical matrix, it provides a chemically grounded external benchmark. Despite these contextual differences, the projection clearly supports the latent structure diagnostics by demonstrating stronger alignment of the standard with LV1 in OSC-pretreated models (Fig. 3C, D). This suggests that the structural reorganization introduced by OSC indeed concentrates analyte-relevant variance on the prime LV and enhances its separation from matrix variance.

This demonstrates that while NAS-LOD is more directly affected by changes in analyte delineation, variance-based mLOD is not structurally blind. In the example (N-500, vitexin, intact; Table 7), the OSC improves both signal isolation and PLS-R latent structure (i.e., calibration geometry). Although by principle less structurally responsive, mLOD exhibits sensitivity to model reorganization and responds significantly to its realignment. Both NAS-LOD and mLOD provide standardizable diagnostic information on the internal structure of the model, albeit in framework-specific ways. Consequently, both LOD frameworks are structurally responsive and provide complementary, not redundant, diagnostic insights into model quality.

#### Diagnostic role of LOD behaviour for model structure quality

These results demonstrate that the LOD frameworks, beyond their quantitative utility for method validation, provide valuable diagnostic insight into model structure and analyte signal integrity. Low NAS-LOD values consistently indicated strong analyte delineation, concentrated variance in early LVs, and high model interpretability—all characteristics in alignment with good-practice PAT expectations. Elevated NAS-LOD, by contrast, reliably exposed models with fragmented or poorly isolated analyte signal, even when conventional error metrics (e.g., RMSEP) or variance-based mLOD suggested acceptable performance. These cases revealed that NAS-LOD responds to geometric analyte alignment within the latent structure, and therefore detects weaknesses not apparent from global error alone. Variance-based mLOD, in contrast, remained less sensitive to such structural dispersion, responding instead to the general geometric relationship between low-concentration samples and the overall latent space constructed in the model. In other words, mLOD does not provide a clear insight into method interpretability. As discussed earlier, this explains why models with similar mLOD but widely differing NAS-LOD may exhibit meaningful differences in analyte interpretability, providing useful metrics for method assessment at a deeper level.

In summary, the combined evaluation of LOD frameworks on the background of spectrometer types (benchtop, miniaturized), analytes (vitexin, isovitexin, joint), and model complexities revealed consistent diagnostic trends. Variance-based mLOD estimates were generally more stable than NAS-LOD across instruments and analytes, but showed substantial responsiveness to structural reorganization in select models. Conversely, NAS-LOD reflects analyte signal concentration and serves as a general indicator of method interpretability. Benchtop spectrometer (N-500) consistently achieved lower detection limits than miniaturized spectrometers, with vitexin models generally outperforming isovitexin, particularly for NAS-LOD. Models quantifying the sum of vitexin and isovitexin exhibited intermediate detection behaviour. Model complexity had opposing effects on the two frameworks; increasing it lowered NAS-LOD but increased mLOD, reflecting the fundamental distinction between analyte-specific variance recovery and global calibration variance. OSC systematically improved both frameworks by compressing model variance and enhancing analyte isolation, with a stronger effect on NAS-LOD. Future studies should focus on developing direct interpretation approaches that link latent space structure with LOD framework-specific detection behaviour, enabling more transparent, standardized calibration diagnostics in phytopharmaceutical applications of NIR spectroscopy.

## Conclusions

Robust development of an NIR-based quantitative method in PAT demands more than accurate prediction alone. This domain particularly emphasizes the transparency and interpretability of the analytical method, as well as a thorough understanding of how analyte information is captured and structured within it. The present study demonstrates that multivariate LOD/LOQ estimation frameworks, when properly selected and interpreted, provide valuable insight into method properties. Variance-based mLOD and NAS-LOD offer fundamentally different diagnostic metrics for multivariate method development: the former assesses global predictive sensitivity, while the latter specifically evaluates the degree of analyte isolation within the latent structure of the model. Their divergence, particularly in complex matrices such as phytopharmaceutical products, demonstrates the need for multi-layered evaluation of the analytical method.

Furthermore, specific steps in method development, such as orthogonalization-based preprocessing, critically influence how analyte variance is structured within the latent variable space. This structural organization significantly impacts detection capability in multivariate calibration, as it is inherently conditional upon analyte representation and variance isolation within the model. NAS-LOD is a framework particularly indicative of this structural organization, directly responding to how analyte variance is isolated and aligned within the model, while variance-based mLOD predominantly reflects global predictive sensitivity of the model, less dependent on internal variance distribution. Multivariate LOQ in both frameworks is proportional to LOD values; thus, it does not provide additional structural diagnostics in modelling beyond that already captured by the LOD framework. However, it is indicative for operational thresholds in practical method implementation. Thus, LOD/LOQ assessment in PAT should not be viewed solely as a validation metric. Instead, it serves as a powerful diagnostic tool that can be readily standardized for evaluating and refining multivariate methods, explicitly revealing how, where, and to what extent analyte variance is captured and delineated within predictive models.

## Supplementary Information

Below is the link to the electronic supplementary material.

Supplementary Material 1 (DOCX 545 KB)

## Data Availability

NIR spectra, reference concentrations, and MATLAB scripts for LOD/LOQ are available upon reasonable request.

## Declarations

### Animal welfare/source of biological material

Not applicable (plant materials from commercial suppliers; see the “Samples” section).
