# Supplementary material for: Standardizing NIR spectroscopy for PAT in phytopharmaceutical applications: multivariate detection and quantification limits of vitexin and isovitexin
Source: Anal Bioanal Chem. 2025 Nov 21;418(1):151–65. doi: 10.1007/s00216-025-06209-z (PMC12774942; doi:10.1007/s00216-025-06209-z)
Supplement: Supplementary file 1 — Supplementary Material 1 (DOCX 545 KB) [file 216_2025_6209_MOESM1_ESM.docx]

**Standardizing NIR spectroscopy for PAT in phytopharmaceutical applications: multivariate detection and quantification limits of vitexin and isovitexin**

*Krzysztof B. Beć^[[1]](#footnote-1)^*, Justyna Grabska^1^, Jan-Clemens Cremer^1^, Christian W. Huck^1^**

*^1^Institute of Analytical Chemistry and Radiochemistry, University of Innsbruck, Innsbruck, Austria*

**Electronic Supplementary Material**


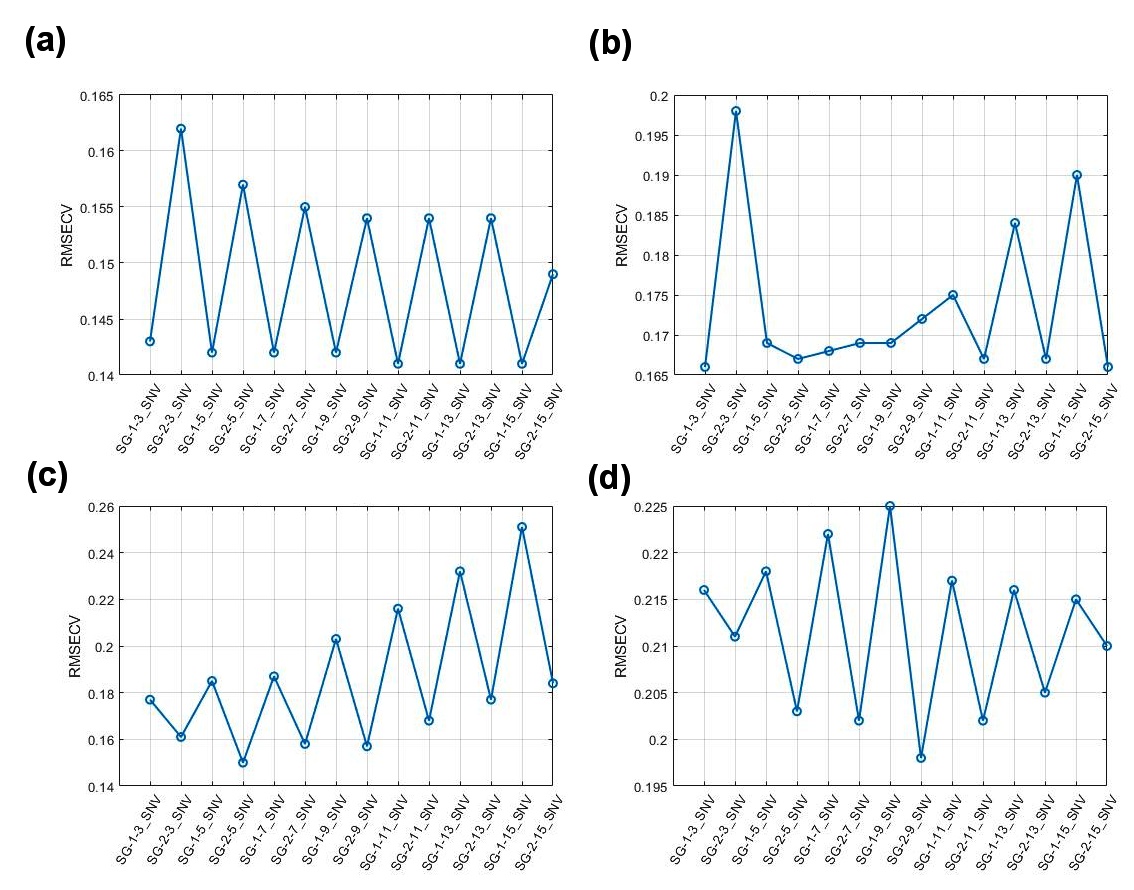


**Fig. S1.** **Model performance (RMSECV) by pretreatment. Panels: (a) Büchi N-500, milled; (b) MicroNIR 2200, milled; (c) MicroNIR 1700 ES, milled; (d) MicroNIR 1700 ES, intact. Each point corresponds to SG derivative order {1, 2} and window size {5–15} (polynomial order = 2), followed by SNV.**


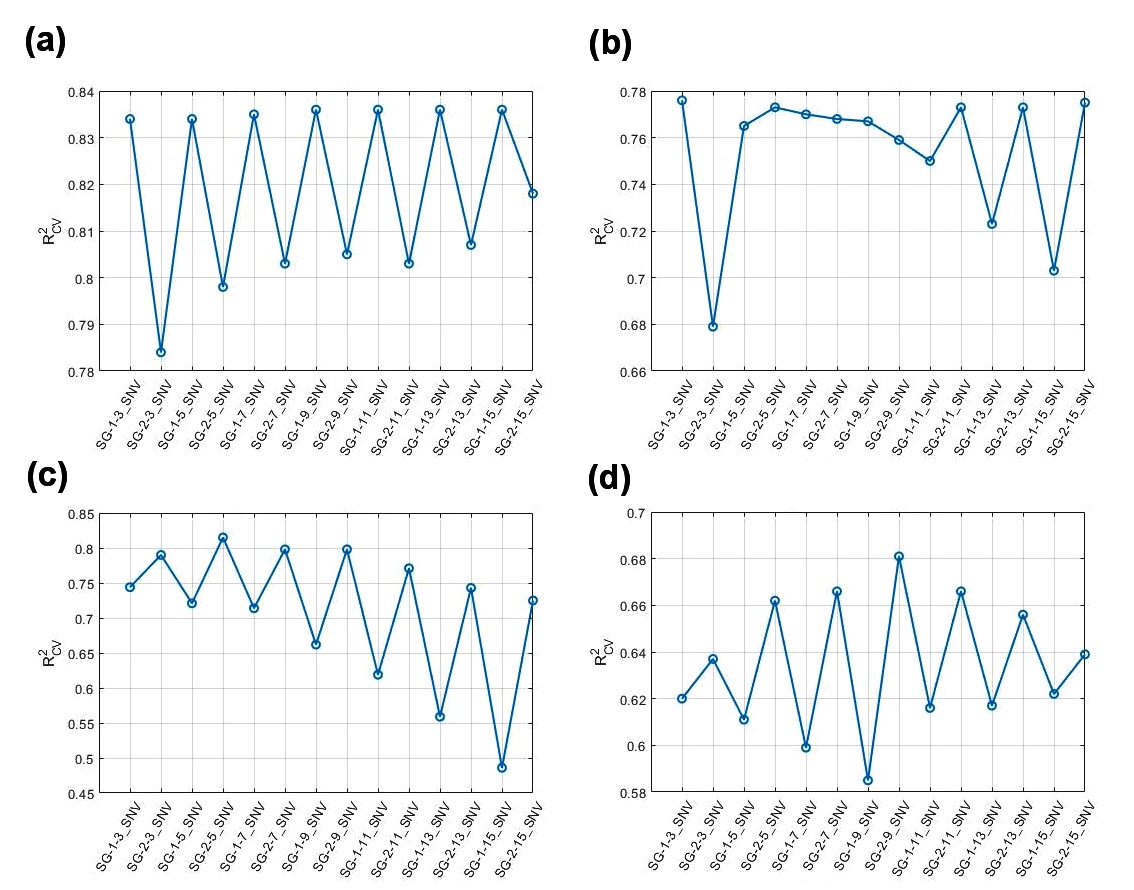


**Fig. S2. Model performance (*R*^2^_CV_) by pretreatment. Panels: (a) Büchi N-500, milled; (b) MicroNIR 2200, milled; (c) MicroNIR 1700 ES, milled; (d) MicroNIR 1700 ES, intact. Each point corresponds to SG derivative order {1, 2} and window size {5–15} (polynomial order = 2), followed by SNV.**

1. * Corresponding authors: [Krzysztof.Bec@uibk.ac.at](mailto:Krzysztof.Bec@uibk.ac.at) (K.B.B.), [Christian.W.Huck@uibk.ac.at](mailto:Christian.W.Huck@uibk.ac.at) (C.W.H.). [↑](#footnote-ref-1)
